# Inflammation-Independent Antinociceptive Effects of DF2755A, a CXCR1/2 Selective Inhibitor: A New Potential Therapeutic Treatment for Peripheral Neuropathy Associated to Non-Ulcerative Interstitial Cystitis/Bladder Pain Syndrome

**DOI:** 10.3389/fphar.2022.854238

**Published:** 2022-04-28

**Authors:** Laura Brandolini, Andrea Aramini, Gianluca Bianchini, Anna Ruocco, Riccardo Bertini, Rubina Novelli, Patrizia Angelico, Anna Elisa Valsecchi, Roberto Russo, Vanessa Castelli, Annamaria Cimini, Marcello Allegretti

**Affiliations:** ^1^ Research and Early Development, Dompé Farmaceutici S.p.A., L’Aquila, Italy; ^2^ Research and Early Development, Dompé Farmaceutici S.p.A., Naples, Italy; ^3^ Atreius S.a.s., L’Aquila, Italy; ^4^ Research and Early Development, Dompé Farmaceutici S.p.A., Milan, Italy; ^5^ Recordati S.p.A., Milan, Italy; ^6^ Department of Pharmacy, University of Naples Federico II, Naples, Italy; ^7^ Department of Life, Health and Environmental Sciences, University of L’Aquila, L’Aquila, Italy

**Keywords:** CXCR1/R2 receptor inhibitor, DF2755A, interstitial cystitis (IC)/bladder pain syndrome (BPS), peripheral neuropathic pain, cyclophosphamide

## Abstract

Interstitial cystitis (IC)/bladder pain syndrome (BPS) is a chronic bladder disease of unknown etiology characterized by urinary frequency and episodic and chronic pain. Analgesic treatments for IC/BPS are limited, especially for patients with non-Hunner (non-ulcerative) type IC who usually have poor overall outcomes. Here, we demonstrate that oral treatment with DF2755A, a potent and selective inhibitor of chemokine receptors CXCR1/2, can prevent and reverse peripheral neuropathy associated to non-Hunner IC/BPS by directly inhibiting chemokine-induced excitation of sensory neurons. We tested DF2755A antinociceptive effects in a cyclophosphamide (CYP)-induced non-ulcerative IC rat model characterized by severe peripheral neuropathy in the absence of bladder inflammatory infiltrate, urothelial hyperplasia, and hemorrhage. Treatment with DF2755A prevented the onset of peripheral neuropathy and reversed its development in CYP-induced IC rats, showing a strong and long-lasting anti-hyperalgesic effect. *Ex vivo* and *in vitro* studies showed that DF2755A treatment strongly inhibited the expression of CXCR2 agonists, CXCL1/KC, and CXCL5 and of transient receptor potential vanilloid 1 (TRPV1) compared to vehicle, suggesting that its effects can be due to the inhibition of the nociceptive signaling passing through the CXCL1/CXCR1-2 axis and TRPV1. In conclusion, our results highlight the key pathophysiological role played by the CXCL1/CXCR1-2 axis and TRPV1 in the onset and development of peripheral neuropathy in non-Hunner IC and propose DF2755A as a potential therapeutic approach for the treatment of not only inflammatory painful conditions but also neuropathic ones and in particular non-Hunner IC/BPS.

## 1 Introduction

Interstitial cystitis (IC)/bladder pain syndrome (BPS) is a multifactorial clinical condition characterized by urinary frequency and urgency and debilitating episodic and chronic pain (Lukban et al., 2001), in the absence of proven urinary infection or other pathologies (Vas et al., 2014; van de Merwe et al., 2008). Two subsets of IC/BPS have been defined based on bladder immunohistochemical characteristics: Hunner-type/classic IC (ulcerative IC), which is an inflammatory disorder characterized by the presence of Hunner lesions (ulcers), and the non-Hunner type IC (non-ulcerative IC), which presents no inflammatory infiltrate and/or hemorrhage (Maeda et al., 2015). Although intensive research has been focused on the identification of therapeutic options for IC/BPS, available treatments often give scarce results, especially in patients affected by the non-ulcerative (non-Hunner) subtype (Whitmore et al., 2019), which is the most prevalent among the two forms of IC (Augé et al., 2020).

To reproduce the features of IC subtypes and to investigate pathways and mediators involved in the pathophysiology of the disease, different animal models have been developed. Some of these involve the administration of cyclophosphamide (CYP), an alkylating agent mainly used for the treatment of neoplasms (Colvin, 2003), which is widely used in rodents to induce experimental IC/BPS (Lehmann and Wennerberg, 2021). In the acute model, single CYP intraperitoneal injection (150–200 mg/kg) induces massive urinary bladder inflammation, visceral pain, and tissue hemorrhage, which are characteristics of the ulcerative form of human IC (Augé et al., 2013); otherwise, rats receiving multiple injections of lower doses of CYP show little or no inflammatory cell infiltration in the bladder, resembling the features of chronic non-ulcerative human IC/BPS (Augé et al., 2020). In this latter chronic model, repeated CYP administration (25–100 mg/kg each dose) induces peripheral neuropathy in rats, which is characterized by abdominal pain associated with increased mechanical sensitivity in the hind paws and abdominal area, indicative of a central sensitization (Augé et al., 2020; 2021) and similar to the neuropathic pain behavior observed in IC/BPS patients (Fariello and Moldwin, 2015).

Experimental models have been instrumental so far for the investigation of the underlying mechanisms and the factors involved in the pathophysiology of IC/BPS. Among IC/BPS mediators, chemokines and their receptors (CXCRs) have attracted the interest of researchers because of their crucial role in both inflammation and nociception (Gonzalez et al., 2014). Chemokines are small secretory molecules that are involved in numerous biological functions, including leukocyte migration and activation, cell adhesion, and T-cell activation (Hughes and Nibbs, 2018). In patients with ulcerative IC/BPS, increased expression of chemokines, including CXCL1-, CXCL10-, and CXCR3-binding chemokines (CXCL9 to CXCL11), was found in bladder interstitium (Ogawa et al., 2010) and in the urine (Tyagi et al., 2012). This resembled what has been observed in the acute model of CYP-induced ulcerative IC, in which inflammation increased the expression of several chemokines, including CX3CL1, CXCL1, CXCL5, CCL2, CXCL10, CXCL12, and their corresponding receptors, in the urinary bladder and in urine (Yuridullah et al., 2006; Sakthivel et al., 2008; Vera et al., 2008; Smaldone et al., 2009; Arms et al., 2010). Notably, the blockade of CXCL10 and CXCL12 signaling reduced the severity of bladder inflammation and hyperexcitability observed in the acute model of CYP-induced ulcerative IC (Sakthivel et al., 2008; Arms et al., 2010), and recent studies on this model have highlighted in particular the role of chemokine receptor 2 (CXCR2) in the mediation of bladder inflammation, overactivity, and nociceptive behavior (Dornelles et al., 2014). Interestingly, in the same model, CXCR2 and transient receptor potential vanilloid 1 (TRPV1), a factor known to be crucial for pain modulation in IC/BPS pathophysiology (Z.Y. Wang et al., 2008b), were found to be markedly expressed in nerve fibers innervating detrusor muscle (Dornelles et al., 2014).

In addition to chemokines’ nociceptive effects as a result of immune system activation, and in particular of their stimulation of leukocyte release of nociceptive mediators (White et al., 2007), growing evidence points to a direct effect of chemokines on pain modulation (Gonzalez et al., 2014). Electrophysiological and functional pain studies have shown in fact that chemokines can directly stimulate neuronal hypersensitivity (Oh et al., 2001; Sun et al., 2006) and both central and peripheral sensitization, thus suggesting that these molecules and their receptors can be potential therapeutic targets for the treatment of not only inflammatory but also neuropathic pain (Piotrowska et al., 2019). Several studies have demonstrated that CXCR2 expression and activation in the nociceptive nervous system play a direct pro-nociceptive role in inflammatory and neuropathic pain modulation. For instance, intraplantar injection of complete Freund’s adjuvant (CFA) increased CXCL1 and CXCR2 protein expression in mice spinal cord and dorsal root ganglion (DRG), while intrathecal injection of CXCL1-neutralizing antibody, CXCR2 antagonist, or perisciatic nerve injection of CXCR2 siRNA attenuated CFA-induced mechanical and heat hypersensitivity (Cao et al., 2014; 2016). In addition, experimental chronic constriction injury of the sciatic nerve raised spinal levels of CXCR2 and CXCR2 agonists, whereas intrathecal administration of CXCL3- or CXCL1-neutralizing antibody inhibited neuropathic pain (Manjavachi et al., 2014; Silva et al., 2017; Piotrowska et al., 2019).

Given the emerging role of chemokines and their receptors in neuropathic pain modulation, we thus sought to investigate whether the inhibition of chemokine receptors CXCR1/2 could reduce pain also in a non-inflammatory setting of IC/BPS, such as the non-ulcerative IC. To this end, we evaluated the potential antinociceptive effects of DF2755A (Colella et al., 2018), which is a potent and highly selective inhibitor of both the CXCL receptor subtypes (Lopes et al., 2016), CXCR1 (IC_50_ = 4.2 nM) and CXCR2 (IC_50_ = 2.1 nM), in a chronic model of CYP-induced urinary bladder painful syndrome. Our results showed that DF2755A can prevent and reverse non-ulcerative IC-associated peripheral neuropathy and that its antinociceptive effects are independent from the modulation of the immune response in this model.

## 2 Materials and Methods

### 2.1 Drugs

DF2755A (sodium (2S)-2-(4-{[4-(trifluoromethyl)-1,3-thiazol-2-yl]amino}phenyl)propanoate) was synthetized by Dompé Farmaceutici S.p.A, L’Aquila, Italy (Lopes et al., 2016). [^14^C]DF2755A was synthetized by Quotient Bioresearch Ltd. with a radiochemical purity of 98.0% and a specific activity of 46 mCi mmol^−1^. The material was supplied as a solution in water in borosilicate multidose vials with an additional screw cap. Cyclophosphamide (CYP) was purchased from Sigma-Aldrich and dissolved in distilled water.

### 2.2 Animals

Female Sprague–Dawley rats (Crl:CD (SD)BR) were used in this study (supplied by Charles River Laboratories Ltd. Margate, Kent, United Kingdom or Charles River Srl, Calco, Lecco, Italy) with a weight range of 200–350 g and age of approximately 7–15 weeks at the time of dosing. Sprague–Dawley has been recurrently chosen as a strain for IC/BPS preclinical modeling (Augé et al., 2020; Chen et al., 2020; Luo et al., 2020), and female animals have higher clinical translational potential given the higher incidence of IC/BPS in women than men (Clemens et al., 2005; Patnaik et al., 2017). The animals were housed up to four in standard polypropylene rat cages (380 × 270 × 200 mm; ∼1,025 cm^2^) at the following environmental conditions: temperature ca. 21 ± 2°C; relative humidity 55 ± 10%; daily light cycle 12-h fluorescent lighting and 12-h dark; and temperature and relative humidity continuously recorded throughout the study. Food and water were available *ad libitum* throughout the study. Rats were killed under CO_2_ anesthesia by decapitation. Animal studies are reported in compliance with the ARRIVE guidelines (Percie du Sert et al., 2020). All animal care and experimental procedures were performed in the animal facilities according to ethical guidelines for the conduction of animal research (Authorization from the Italian Ministry of Health N. 271/95-B DL 116/92; Gazzetta Ufficiale della Repubblica Italiana N. 40, 18 February 1992; EEC Council Directive 86/609 OJ L 358, 1 12 December 1987; Directive 2010/63/EU; NIH Guide for the Care and Use of Laboratory Animals, NIH Publication N. 85–23, 1985) and were approved by the Local Animal Ethics Committee (CETEA, UFMG; Protocol Number: 147/06).

### 2.3 Pharmacokinetic Studies

Pharmacokinetics of DF2755A, dissolved in saline solution (NaCl 0.9% w/v), was investigated after single intravenous and oral administrations in male Wistar Han rats. DF2755A was given as a single dose in three animals/group by intravenous (bolus) and oral (by gavage) administrations at a dose of 20 mg/kg. Pharmacokinetic evaluation was carried out on blood samples collected on day 1 at six time points from three animals/group (serial sampling).

### 2.4 Selectivity

Selectivity assays were performed at Eurofins Cerep as a contract service. A summary of the protocol and the reference for the assays are listed on the Eurofins Cerep website at (https://www.eurofinsdiscoveryservices.com/catalogmanagement/viewItem/SafetyScreen44-Panel-Cerep/P270). The tested targets are listed below:

GPCRs: A2A (agonist radioligand), α1A (antagonist radioligand), α2A (antagonist radioligand), β1 (agonist radioligand), β2 (agonist radioligand), BK1 (antagonist and agonist radioligand), BK2 (antagonist and agonist radioligand), CB1 (antagonist and agonist radioligand), CB2 (antagonist and agonist radioligand), CCK1 (CCKA) (agonist radioligand), D1 (antagonist radioligand), D2 (antagonist and agonist radioligand), D3 (antagonist and agonist radioligand), ETA (agonist radioligand), H1 (antagonist radioligand), H2 (antagonist radioligand), M1 (antagonist radioligand), M2 (antagonist and agonist radioligand), M3 (antagonist radioligand), NK1 (agonist radioligand), δ (DOP) (agonist radioligand), κ(KOP) (agonist radioligand), µ(MOP) (agonist radioligand), ORL1 (agonist radioligand), 5-HT1A (agonist radioligand), 5-HT1B (antagonist radioligand), 5-HT2A (agonist radioligand), 5-HT2B (agonist radioligand), and V1a (agonist radioligand).

Transporters: 5-HT transporter (antagonist radioligand), dopamine transporter (antagonist radioligand), and norepinephrine transporter (antagonist radioligand).

Ion channels: 5-HT3 (antagonist radioligand), BZD (central) (agonist radioligand), NMDA (antagonist radioligand), N neuronal alpha 4 beta 2 (agonist radioligand), Ca^2+^ channel (L-dihydropyridine site) (antagonist radioligand), Na^+^ channel (site 2) (antagonist radioligand), and KV (antagonist radioligand).

Nuclear receptors: AR (agonist radioligand) and GR (agonist radioligand).

Kinase and other non-kinase enzymes: CTK Lck kinase, acetylcholinesterase, PDE3A, PDE4D2, and MAO-A (antagonist radioligand).

In addition, DF2755A was tested on TRPM8, TRPV1, TRPV4, TRPA1, and Nav1.7 ion channels in agonist and antagonist mode at eight concentrations in quadruplicate (100 μM was the highest tested concentration).

### 2.5 Excretion and Tissue Distribution Studies Following Single Oral Administration of [^14^C]DF2755A

The routes and rates of excretion together with the tissue distribution were investigated following a single oral administration of radiolabeled [^14^C]DF2755A at a target dose of 20 mg kg^−1^.

Prior to administration and for acclimatization, animals (*n* = 6) were transferred to individual metabolic cages and were left therein for the duration of the study except for a short period during dosing. The metabolic cages were specifically designed for the separate collection of urine and feces. The preparation of dose formulation was performed on the day of experiment. The test item was dissolved in the appropriate volume of saline solution (NaCl, 0.9% w v^−1^) to achieve the dose volume of 5 ml kg^−1^. The resulting solution was then administered *via* a gavage fed down the esophagus to enable the formulation to be dispensed directly into the stomach. For the pharmacokinetic analysis of the radiolabeled drug, six female SD rats received a single oral administration of [^14^C]DF2755A at a target dose of 20 mg free-compound/kg. Whole blood was collected at 0.25, 0.5, 1, 2, 4, 6, 8, 10, 12, 24, 28, 32, 36, 48, and 168 h post-administration, and mean concentrations of total radioactivity in plasma were determined.

At 168 h after administration, the animals were removed from the metabolism cage and killed by cervical dislocation. Radioactivity was extracted from the selected feces and urine homogenate pools with appropriate solvents based on methodology developed at Quotient (Rushden) Ltd. and analyzed by quantitative radioactivity analysis (QRA). Tissue distribution was performed by quantitative whole-body autoradiography (QWBA). At the selected time points (2, 6, 12, 24, 48, 120, and 168 h post-oral administration of the compound), animals (n = 6) were killed using an overdose of carbon dioxide, followed by immediate snap freezing in a hexane/solid carbon dioxide mixture. Each carcass was subjected to WBA using procedures based on the work of Waddell and Brinkhous (1967). Sections were taken at up to six different levels of the rat body to include as many tissues as possible (a minimum of 30). Distribution of radioactivity in the sections was determined and quantified by bioimage analysis.

### 2.6 Chronic Model of Non-ulcerative CYP-Induced IC/PBS

CYP was administered to the animals by the intraperitoneal (i.p.) route (5 ml kg^−1^) at the dosage of 75 mg kg^−1^ for three times every third day to elicit chronic bladder irritation, as previously described (Vizzard et al., 1996).

DF2755A was administered by oral gavage (2 ml kg^−1^) at different doses and times before, during, or after CYP treatment, as specified hereunder. DF2755A dosages were chosen based on previous studies that described pharmacokinetics, pharmacodynamics, and pharmacological characterization of the compound (Lopes et al., 2016).

Acute treatment: DF2755A was administered 24 h after the last CYP administration at 3, 7, 10, and 30 mg kg^−1^.

Chronic preventive treatment: DF2755A was administered 12 h before the first CYP administration (20 mg kg^−1^) and then twice daily (7 mg kg^−1^) for 7 days during IC development.

Chronic therapeutic treatment: DF2755A was administered twice daily for 7 days at 7 mg kg^−1^, starting from 24 h after the last CYP injection.

### 2.7 Mechanical Nociceptive Abdominal and Hind Paw Test

On the day of the test, each rat was placed individually in a clear plastic testing box with a grid floor and allowed to acclimatize for at least 10 min. Peripheral sensitivity in response to mechanical stimuli was determined using Von Frey monofilaments (1-2-4-8-15-26-60–100 g; Ugo Basile, Comerio, Varese, Italy) applied in the lower abdominal area (close to the urinary bladder) and on both hind paw plantar surfaces (to evaluate referred pain).

Each Von Frey monofilament was applied five times at the level of abdomen and three times at the level of each paw, in an ascending order of strength at interval of 5 s. A stimulus-induced response was considered positive when the paw was sharply withdrawn, paw licking occurred, or the animal flinched upon removal of the filament.

Behavioral testing was performed at different times:

1) before CYP administration, in order to acquire basal values;
2) after CYP administration and before DF2755A treatment, in order to verify the pathology induction and acquire pre-treatment values;
3) at different times after DF2755A treatment, in order to acquire post-treatment values, specifically at 2 and 18 h after the last treatment.

### 2.8 Bladder Tissue Samples Preparation

Animals pre-treated with the vehicle or a single high oral dose of DF2755A (20 mg kg^−1^; 12 h before the first CYP administration) followed by repeated lower doses administration (7 mg kg^−1^ twice daily for 7 days) during IC development were deeply anesthetized with isoflurane (4%) and then killed.

After killing, rat abdomen was opened through a midline incision, and bladder and proximal urethra were exposed. A catheter was inserted into the bladder through the urethra and a loose ligature was placed around the neck of the urinary bladder. Bladder was inflated with saline *via* the catheter to slightly distend the bladder and ligature was tightened to maintain proper distension. Bladder was then excised cutting above the insertion point of ureters, and therefore the bladder neck was not included into the bladder sample. Bladders were opened using scissors and, after gross macroscopic evaluation and weighing, each bladder was cut into two equal pieces (named B1 and B2) and weighted again individually. One piece (B1) was placed in Eppendorf, then snap-frozen in liquid nitrogen, and stored at −80°C until quantification using ELISA assay. The second piece (B2) was placed in 10% formalin and stored at room temperature. Then the same samples were placed in 70% ethanol, then processed in paraffin blocks, and then trimmed according to the longitudinal plane to ensure the correct analysis of all the bladder sample. Sections were stained with hematoxylin and eosin (HE), and slides were then digitalized using a Pathscan^®^ Touch scanner in the bright field mode with a ×20 objective. For the microscopic observations of bladder inflammation, the following parameters were analyzed: bladder edema in scores (*n* = 10/group), bladder hemorrhage in scores (*n* = 10/group), urothelial loss in scores (*n* = 10/group), and total inflammatory score, which was calculated by adding all the individual lesion scores (*n* = 10/group). These parameters were scored according to the grading scheme reported in Table 1(Augé et al., 2020).

**TABLE 1:** Grading scheme used for bladder tissue histological evaluation.

Score	Edema	Hemorrhage	Urothelial loss
0	Normal	Normal	Normal
1	Minimal	Minimal	Focal
2	Mild	Mild	Multifocal
3	Moderate	Moderate	Extensive
4	Marked	Marked	Diffuse

### 2.9 CXCR2-Agonist Expression in Bladder Tissue and Histopathologic Analysis

Frozen bladder B1 pieces isolated from the end of the study were thawed and individually lysed using ice-cold lysis buffer (20 mM Tris–HCl (pH 7.5), 10 mM NaF, 150 mM NaCl, 1% Nonidet P-40, 1 mM phenylmethylsulfonyl fluoride, 1 mM Na_3_VO_4_, leupeptin, and trypsin inhibitor 10 μg ml^−1^; 0.25/50 mg tissue). Tissues were homogenized using a Polytron homogenizer and centrifuged at 10,000 rpm for 10 min and analyzed by using the Bio-Rad protein assay using bovine serum albumin as standard.

Concentrations of CXCL1 and CXCL5 in bladder tissues were estimated by specific rat ELISA kits (Quantikine; R&D Systems, Minneapolis, MN, United States and MyBioSource Inc., United States), according to the manufacturer’s instructions. The absorbance values of standards and samples from urinary bladder were represented as normalization on µg of protein.

### 2.10 Bladder Tissue Western Blotting Analyses

Protease and phosphatase inhibitors were added to the bladder homogenates extracted. NuPAGE lithium dodecyl sulfate sample buffer (Cat#NP007, Thermo, United States) and NuPAGE sample reducing agent (Cat#NP0004, Thermo, United States) were added to the samples and then put in the Thermoblock (Eppendorf, United Kingdom) at 75°C for 10 min. The extracted proteins were quantified using BCA assay (Thermo, United States). Protein lysates (15 μg) were separated on a NuPAGE bis–tris glycine gradient precast gel (Cat#NP0321PK2, Invitrogen, United States) and electroblotted onto a polyvinyl difluoride membrane (Cat#IPVH00010, PVDF, Millipore, United States) using a semi-dry system (Thermo, United States). Non-specific binding sites were blocked by blocking solution (Cat#000105, Invitrogen, United States) for 15 min at room temperature (RT). Membranes were then incubated overnight at 4°C with the following primary antibodies, diluted in the blocking solution: rabbit horseradish peroxidase–conjugated actin 1:10,000 (Cat#5125, Cell Signaling Technology, United States), rabbit polyclonal IgG anti-transient receptor potential vanilloid (TRPV) 1 1:200 (Cat#PA5-77316, Thermo, United States), and rabbit polyclonal IgG anti-TRPV4 1:200 (Cat#sc-98592, Santa Cruz, United States). As secondary antibodies, peroxidase-conjugated anti-rabbit IgG (1:10,000, Vector, United States) were used. All the antibodies were freshly prepared. Immunoreactive bands were visualized by Pierce Enhanced Chemiluminescence (ECL) Western Blotting Substrate (Cat#32106, Thermo, United States), according to the manufacturer’s instructions. The relative densities of the immunoreactive bands were determined and normalized with respect to actin, using Fiji software. Values were given as relative units.

### 2.11 Cell Culture and Drug Treatment

F11 hybridoma cell line (ECACC 08062601), chosen as a model of dorsal root ganglion (DRG) neurons (Platika et al., 1985; Brandolini et al., 2017) was cultivated in DMEM (Corning, United States) supplemented with 10% heat-inactivated FBS (Sigma-Aldrich St. Louis, CO, United States), 1% penicillin/streptomycin, and 1% glutamine (Sigma-Aldrich, United States) at 37°C, in a humidified 95% air and 5% CO_2_ atmosphere. For these experiments, cells at 18th passage were used. Cells were differentiated with the rat nerve growth factor (rNGF; Sigma-Aldrich, United States). rNGF was dissolved in DMEM with 1% penicillin/streptomycin and 1% glutamine (FBS free) at the final concentration of 50 ng ml^−1^. Medium was replaced every 3 days until complete differentiation, which occurred after 7 days.

Following differentiation, neurons were treated for 24 h with DF2755A 0.1–5 μM or CYP 20–200 nM. Cell viability was determined using the colorimetric method CellTiter AQueous One Solution Cell Proliferation Assay (Promega Corporation, United States), as previously reported (Brandolini et al., 2017). The quantity of formed formazan, as a function of viability, was measured at 492 nm using an ELISA plate reader, Infinite F200 (Tecan, Männedorf, Swiss). The results from five independent experiments were expressed as absorbance at 492 nm.

### 2.12 RNA Isolation and Quantitative Real-Time PCR

TRPV1 and TRPV4 mRNA expression and protein levels and CXCR1, CXCR2, and CXCL1 mRNA levels were evaluated in F11 cells exposed to CYP. After differentiation, cells were treated with DF2755A 1 μM or CYP 100 nM or the combination of the two molecules for 24 h. Total RNA was extracted by using a TRIzol reagent (Life Technologies, United States), according to the manufacturer’s instructions after 6 h of treatment. The total RNA concentration was determined in RNAase-free water using Nanodrop, while the concentration was determined using the Qubit Fluorometer 3.0 (Thermo, United States). Total RNA of 1 μg was reverse transcribed using a 5X All-In-One RT MasterMix (Applied Biological Materials, Canada) into cDNA using Thermoblock (Eppendorf, United Kingdom). Finally, the real-time PCR was carried out on using the ABI 7300HT sequence detection system (ABI), containing 2X TaqMan Gene Expression Master Mix (Invitrogen, United States), DEPC water, 5 μl of cDNA, and 1 μl the following primers: Prime Time qPCR Assay: TRPV1 qRnoCIP0024978, TRPV4 qRnoCIP0027857, CXCR1 Mm 99999117_s1, CXCR2 Mm 00731329_s1, and CXCL1 Mm 04207460_m1 were purchased from Bio-Rad, United States. Triplicate samples were run for each gene. The reference gene GAPDH qRnoCIP0050838 was used as an internal control to normalize the expression of TRPV1 and TRPV4 genes, while GAPDH Mm99999915_g1 was used as the internal control to normalize the expression of CXCR1, CXCR2, and CXCL1. Relative expression levels were calculated for each sample after normalization against the reference gene, using the 2^−ΔΔCt^ method for comparing relative fold expression differences.

### 2.13 Western Blotting Analyses

Control and treated F11 cells were collected and lysed in an ice-cold RIPA buffer containing protease and phosphatase inhibitor cocktail (Sigma-Aldrich, United States). The extracted proteins were quantified using BCA assay (Thermo, United States). NuPAGE lithium dodecyl sulfate sample buffer (Cat#NP007, Thermo, United States) and NuPAGE sample reducing agent (Cat#NP0004, Thermo, United States) were added to the samples and then put in the Thermoblock (Eppendorf, United Kingdom) at 75°C for 10 min. Protein lysates (30 μg) were separated on NuPAGE bis–tris glycine gradient precast gel (Cat#NP0321PK2, Invitrogen, United States) and electroblotted on a PVDF membrane (Cat#IPVH00010, Millipore, United States) using a semi-dry system (Thermo, United States). Non-specific binding sites were blocked by blocking solution (Cat#000105, Invitrogen, United States) for 15 min at RT. Membranes were then incubated overnight at 4°C with the following primary antibodies, diluted in the blocking buffer: rabbit horseradish peroxidase–conjugated actin 1:10,000 (Cat#5125, Cell Signaling Technology, United States), rabbit polyclonal IgG Anti-TRPV1 1:200 (Cat#PA5-77316, Thermo, United States), and rabbit polyclonal IgG anti-TRPV4 1:200 (Cat#sc-98592, Santa Cruz, United States). As secondary antibodies, peroxidase-conjugated anti-rabbit IgG (1:10,000, Vector, United States) were used. All the antibodies were freshly prepared. Immunoreactive bands were visualized by ECL (Bio-Rad, United States), according to the manufacturer’s instructions. The relative densities of the immunoreactive bands were determined and normalized with respect to actin, using Fiji software. Values were given as relative units.

### 2.14 Data and Statistical Analysis

Randomization was used in all *in vivo* experimental sections. Rats were randomized into vehicle and DF2755A-treated groups. Blinding of the operators was feasible in both *in vivo* and *in vitro* studies. Data analysis was performed blinded by an independent analyst.

The number of experiments was selected during experimental design based on considerations not to employ an unnecessary number of animals (3R principles) and to get evidence for an effect of reasonable size in each experimental determination. Based on the experience of previous studies, in excretion and tissue distribution studies, the number of animals in each group was five; conversely, as determination of mechanical nociception can be more variable, in the efficacy studies, the number of animals in each group was 10. The group size is the number of independent values, and statistical analysis was performed using these independent values. Outliers were included in data analysis and presentation. Data are reported as mean ± SEM. Comparisons between two groups were carried out using Student’s *t*-test for paired data. For three or more groups, comparisons were carried out using one-way ANOVA followed by Tukey’s test for multiple comparisons (GraphPad 4 software, San Diego, CA, United States) or two-way ANOVA by S.A.S./STAT software as specified in figure legends. Differences were accepted as statistically significant if the *p* value was less than 0.05.

## 3 Results

### 3.1 Pharmacokinetic and Selectivity Profile of DF2755A

Intravenous administration of 20 mg kg^−1^ of DF2755A showed slow systemic clearance. The volume of distribution at steady state was also below 1 L/kg with respect to total body water. Plasma concentration at time zero (C0) and overall systemic exposure (AUC0-t (last)) were 117,237 ng/ml and 578,017 ng/mlh, respectively. The t_1/2,z_ was 8.1 h after dosing. Oral administration of 20 mg/kg^−1^ of DF2755A showed a maximum plasma concentration (Cmax) and overall systemic exposure (AUC0-t (last)) of 43,500 ng/ml and 497,968 ng/ml h, respectively. The t_max_ occurred at 2 h after dosing. The systemic exposure to DF2755A (as measured by AUC0-t (last) and Cmax) were proportional and increased with the increasing dose. DF2755A showed a high oral absolute bioavailability (F%) of 86.2%, when administered at 20 mg/kg^−1^ in male Wistar Han rats.

To assess its off-target activities, DF2755A was tested at 10 μM toward a panel of GPCRs, enzymes, ion channels, transporters, and nuclear receptors. We first tested the compound in the safety screen assay performed at Cerep Panlabs, Inc. to provide the identification of significant off-target interactions for the optimization of safety margins (Bowes et al., 2012). Results showed neglectable effects of the tested compound on all the receptors studied. In addition, DF2755A was also tested on TRPM8, TRPV1, TRPV4, TRPA1, and Nav1.7 ion channels, showing no activity in both agonism and antagonism mode up to 100 μM that was the maximum tested concentration.

### 3.2 Excretion and Tissue Distribution Studies Following Single Oral Administration of [^14^C]DF2755A to Female SD Rats

Before investigating the effects of DF2755A in non-ulcerative IC/BPS, pharmacokinetics, excretion, and tissue distribution of the compound were evaluated after single oral administration in naïve rats. The obtained pharmacokinetic parameters are summarized in Table 2. Radioactivity distribution studies showed that the mean overall recovery of radioactivity from 0 to 168 h post-dose was 102.06% of dose. Excretion was relatively rapid, with respective overall mean of 97.67% of dose recovered from 0 to 48 h post-dose, and it was predominantly *via* the fecal route, with a mean of 77.10% of dose observed from 0 to 168 h post-dose. Over the same period, the radioactivity recovered in urine accounted for a mean of 24.13% of dose, and negligible concentrations of radioactivity were detected in the cage washings, residual carcass, and expired air (Table 3). Evaluation of radioactivity in tissues at t_max_ (2 h after dosing) revealed that the greatest radioactivity concentrations were in the stomach contents (51.8 μg equivalents DF2755A g^−1^ of tissue), small intestine contents (180 μg equivalents DF2755A g^−1^ of tissue), kidney cortex (33.2 μg equivalents DF2755A g^−1^ of tissue), kidney as a whole (29.6 μg equivalents DF2755 A/g^−1^ of tissue), liver (41.7 μg equivalents DF2755A g^−1^ of tissue), and blood content into the heart (38.3 μg equivalents DF2755A g^−1^ of tissue). Notably, high radioactivity concentrations have been detected in the urinary bladder contents (63.0 μg DF2755A g^−1^ of tissue) and in the urinary bladder wall (24.3 μg equivalents DF2755A g^−1^ of tissue) corresponding to the 8 and 3% of the administered dose, respectively.

**TABLE 2:** Pharmacokinetic parameters following a single oral administration of [^14^C]DF2755A to female SD rats at a target dose of 20 mg free-compound/kg.

Parameter	Radioactivity in plasma
Tmax (h)	2
Cmax (µg equivalents/g))	55.80
AUC0-t (µg equivalents.h/g)	621.625
AUC0-inf (µg equivalents.h/g)	628.266
Rate constant for the terminal elimination phase k (h^−1^)	0.0634
t_½_ (h)	10.934

**TABLE 3:** Routes and rates of excretion following a single oral administration of [^14^C]DF2755A to female SD rats at a target dose of 20 mg free-compound/kg.

Sample (0–168 h)	Mean recovery (% dose)
Urine	24.13
Feces	77.10
Cage wash	0.26
Expired air	0.07
Carcass	0.51
Total	102.06

These data demonstrated that DF2755A has suitable adsorption, distribution, metabolism, and excretion (ADME) characteristics and peculiar tissue distribution features that allow it to reach bladder tissue at pharmacologically active concentrations after oral administration.

### 3.3 Peripheral Neuropathy in Experimental Non-Ulcerative IC/BPS and DF2755A Dose-Response Antinociceptive Effect

**FIGURE 1:**
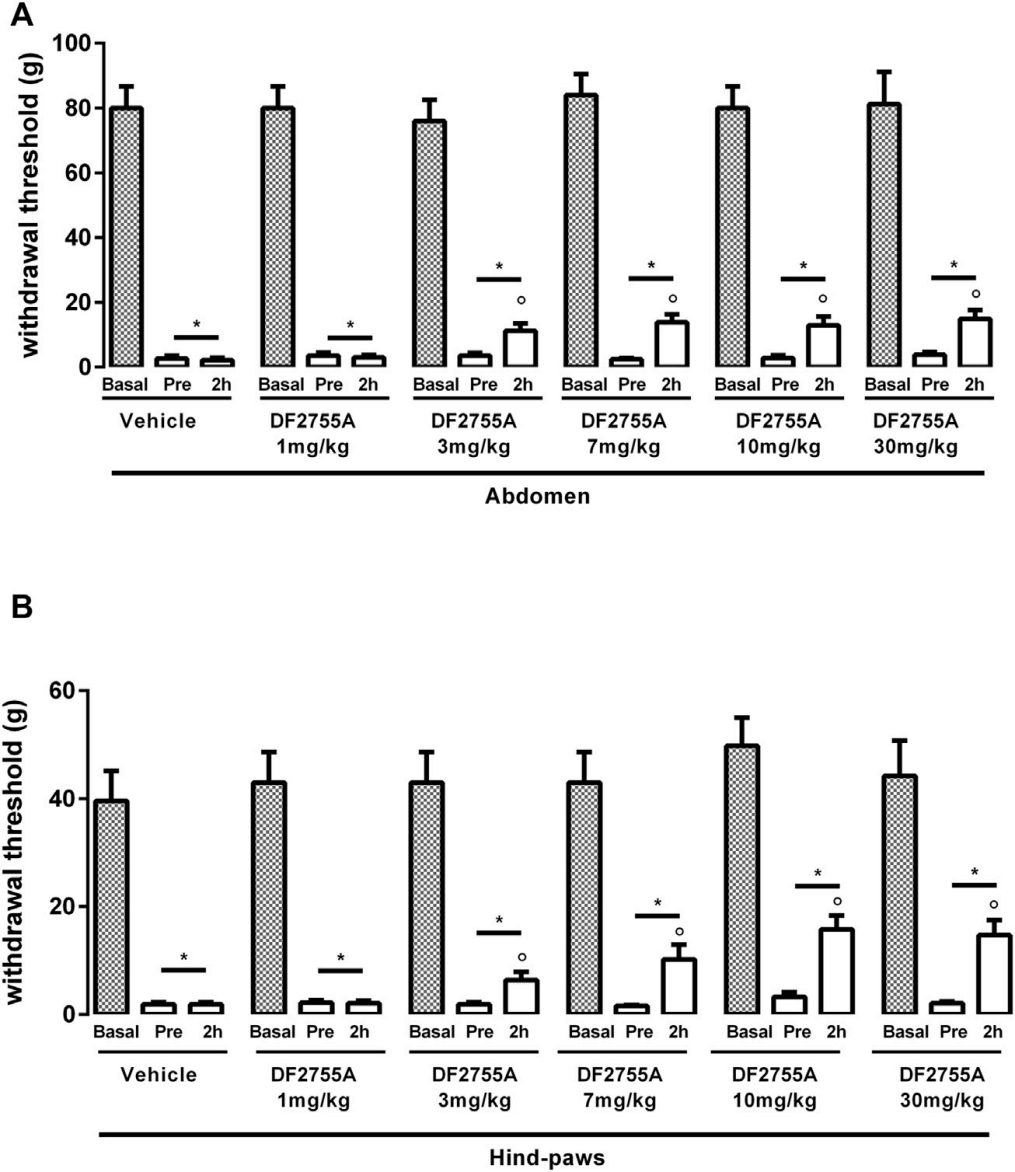
Dose-dependent effect of DF2755A on peripheral pain in experimental non-Hunner IC. After chronic systemic i.p. injection of CYP, rats were treated p.o. with the vehicle or a single dose of DF2755A (1–30 mg kg-1). Mechanical nociceptive threshold was measured on the abdominal area **(A)** or hind paw surfaces **(B)** before the first CYP injection (basal), 24 h after the last CYP injection and before vehicle or DF2755A treatment (pre), and 2 h after vehicle or DF2755A treatment (2 h). Data represent the withdrawal threshold and are expressed as mean values ± SEM (*n* = 10 per group) *p < 0.05 versus basal values; ° *p* < 0.05 versus pre-treatment values (one way ANOVA with Tukey’s test).

**FIGURE 2:**
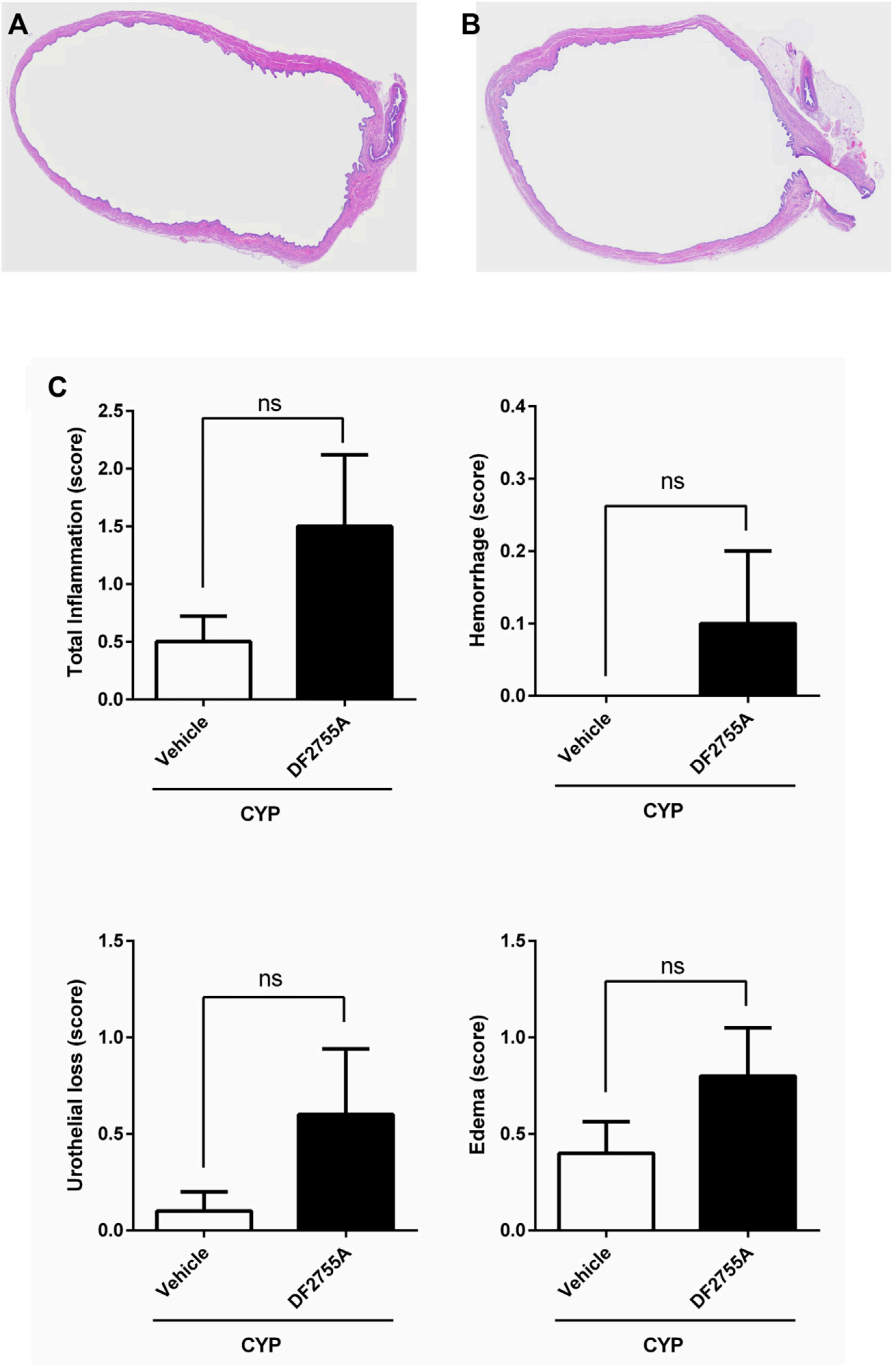
Chronic CYP administration and DF2755A treatment did not affect urinary bladder histology. Rats were orally treated with repeated vehicle or DF2755A (20 mg kg^−1^ 12 h before the first CYP injection +7 mg kg^−1^ twice daily for 7 days) administrations. After 24 h of the last CYP injection, urinary bladders were removed and processed as described in Methods. Sections of hematoxylin and eosin-stained bladder pieces from CYP- **(A)** and CYP +DF2755A-treated animals **(B)** are shown. Scale bar, 200 µm. Total inflammatory score, urothelial loss (score), hemorrhage (score), and bladder edema (score) in CYP rats treated with vehicle or DF2755A **(C)**. Results are expressed as mean ± SEM (*n* = 10 per group). ns *p* > 0.05, Mann–Whitney test.

In line with previous studies (Augé et al., 2020; 2021), repeated injections of CYP-induced peripheral neuropathy in female SD rats, dramatically decreasing the withdrawal thresholds to noxious stimuli measured 24 h after the last CYP administration at the level of low abdomen (Figure 1A) and hind paws (Figure 1B). The histological analysis showed that no inflammatory infiltrate and no urothelial hyperplasia were detectable in rat urinary bladders after chronic CYP administration (Figures 2A,C), thus resembling human non-ulcerative IC (Augé et al., 2020). Similarly, no hemorrhage was observed in this experimental group, while urothelial loss was only present in 1 rat out of 10 leading to a very low mean score (0.1 ± 0.10) comparable to the edema one (0.4 ± 0.16) (Figures 2A,C).

Having established in our hands the CYP-induced non-ulcerative IC rat model, we performed a dose-response experiment in order to identify the dose of DF2755A that was more effective in reducing the peripheral pain. Acute treatment of DF2755A administered 24 h after the last CYP injection inhibited CYP-induced nociceptive responses at both the abdomen (Figure 1A) and hind paw area (Figure 1B) 2 h after the compound administration, and this antinociceptive effect was statistically significant starting from 3 mg kg^−1^. At the dose of 7 mg kg^−1^, the effects seemed to reach a plateau, as no further significant reduction of peripheral pain was reported at higher doses of the compound (10 and 30 mg kg^−1^). As expected, vehicle did not show any effects.

Based on these data, the dose of 7 mg kg^−1^ was chosen for DF2755A chronic treatment for all the subsequent experiments.

### 3.3 DF2755A Prevented Mechanical Nociception in Experimental IC

**FIGURE 3:**
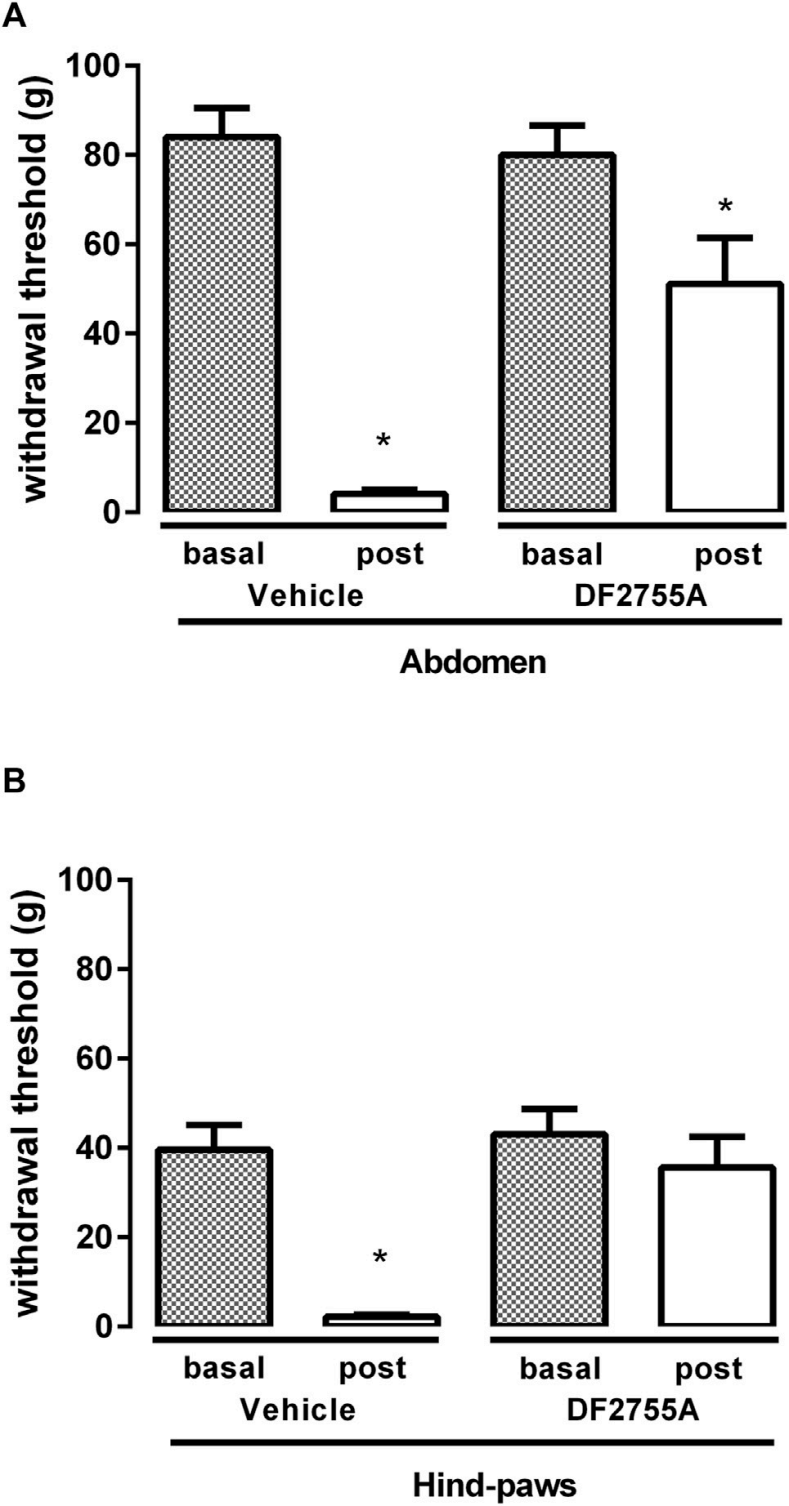
DF2755A preventive treatment blocked peripheral neuropathy in experimental non-Hunner IC. Rats were orally treated with repeated vehicle or DF2755A (20 mg kg^−1^ 12 h before the first CYP injection +7 mg kg^−1^ twice daily for 7 days) administrations. Mechanical nociceptive threshold was measured on the abdominal area **(A)** or hind paw surfaces **(B)** before the first CYP injection (Basal) and 24 h after the last CYP injection (Post). Data represent the withdrawal threshold and are expressed as mean values ± SEM (*n* = 10 per group) *p < 0.05 versus basal values (Student’s t test for paired data).

To investigate whether DF2755A treatment could prevent the onset and development of CYP-induced peripheral neuropathy, DF2755A was administered as preventive chronic treatment 12 h before the first CYP administration (20 mg kg^−1^) and then twice daily (7 mg kg^−1^) for 7 days during IC development. DF2755A significantly prevented CYP-induced peripheral pain: at the level of low abdomen, it blocked the decreasing of mechanical stimulus threshold at about 30% of basal values compared to 95% of threshold reduction that was reached by vehicle-treated animals (Figure 3A), while in hind paws, it blocked the decreasing of the threshold at almost the basal values (17% of decrease) compared to 94% of threshold reduction observed in the vehicle-treated group (Figure 3B). Notably, no significant difference was observed between vehicle- and DF2755A-treated rats for all histological evaluated parameters (Figures 2A–C).

These results clearly demonstrate that DF2755A can prevent the onset and the development of the peripheral neuropathy induced by chronic CYP administration while having no significant effects on bladder inflammation in this model that is characterized by no inflammatory infiltrate.

### 3.4 DF2755A Reversed Peripheral Neuropathy in CYP-Induced IC

**FIGURE 4:**
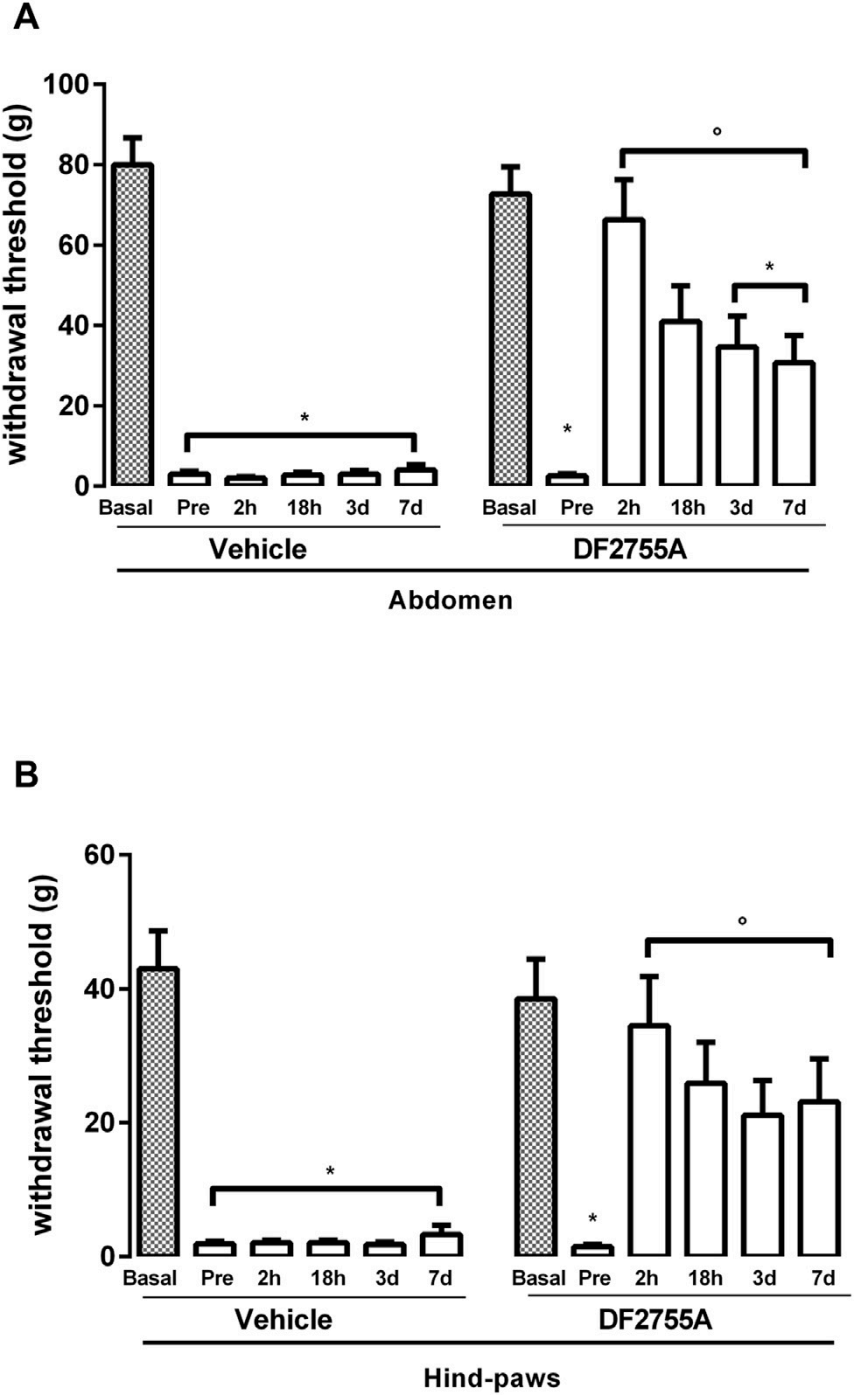
Long-lasting efficacy of DF2755A in reversing peripheral neuropathy in experimental non-Hunner IC. Animals were orally treated with repeated vehicle or DF2755A (7 mg kg^−1^ twice daily for 7 days) administrations starting from 24 h after the last CYP injection. Mechanical nociceptive threshold was measured on the abdominal area **(A)** or hind paw surfaces **(B)** before the first CYP injection (basal), after the last CYP injection and before vehicle or DF2755A treatment (pre), and 2 h (2 h), 18 h (18 h), 3, and 7 days after the last vehicle or DF2755A treatment. Data represent the withdrawal threshold and are expressed as mean values ±SEM (*n* = 10 per group). *p < 0.05 versus basal values; °*p* < 0.05 versus pre-treatment values (two-way ANOVA with Tukey’s test).

Since DF2755A could counteract the onset and the development of CYP-induced IC, we then investigated whether the compound could also exert a therapeutic effect in these conditions. DF2755A was thus administered as therapeutic treatment twice daily for 7 days at 7 mg kg^−1^, starting from 24 h after the last CYP injection. Strikingly, treatment with DF2755A almost completely reversed the decrease of the threshold to mechanical stimuli at the level of low abdomen, passing from 97% reduction observed in rats before the first compound administration and in the vehicle-treated group to 24% reduction observed in the DF2755A-treated group (Figure 4A). Even greater effects were observed at the hind paw plantar surfaces, where the decrease of the threshold to mechanical stimuli was only 4% in DF2755A-treated experimental group, while it was 95% in rats analyzed before the first compound administration and in the vehicle-treated group (Figure 4B).

These data demonstrate the therapeutic effects of the compound that effectively reversed peripheral neuropathy in CYP-induced non-ulcerative IC.

### 3.5 Long-Lasting Anti-Hyperalgesic Effect of DF2755A in CYP-Induced IC

To investigate whether the anti-hyperalgesic effect of DF2755A could last also after treatment discontinuation, we analyzed rat responses to mechanical stimulus at 18 h, 3, and 7 days after the last administration of DF2755A. After 18 h, the therapeutic effect of DF2755A was partially reduced but still highly evident and significant both at the low abdomen level, where a 55% decrease of mechanical stimulus threshold was detected compared to 97% decrease observed in rats analyzed before the first compound administration and vehicle-treated group (Figure 4A), and at the hind paw plantar surfaces, where a 33% decrease of the threshold to mechanical stimulus was detected compared to 97% observed in rats analyzed before the first compound administration and vehicle-treated group (Figure 4B). Interestingly, at 3 and 7 days after treatment discontinuation, DF2755A still exerted a strong and significant therapeutic effect against CYP-induced peripheral neuropathy at the level of both low abdomen, with 53 and 95% reduction of the threshold to mechanical stimulus in the DF2755A-treated group and vehicle-treated animals, respectively (Figure 4A), and hind paw plantar surfaces with 40 and 92% decrease of the threshold to mechanical stimulus in the DF2755A-treated group and vehicle-treated animals, respectively (Figure 4B). At later time points, we observed an overlap of the withdrawal thresholds between vehicle and DF2755A-treated groups, mainly due to the spontaneous recovery of the pathology over the time.

### 3.6 DF2755A Counteracted CYP-Induced Chemokines and TRPV1 Upregulation

**FIGURE 5:**
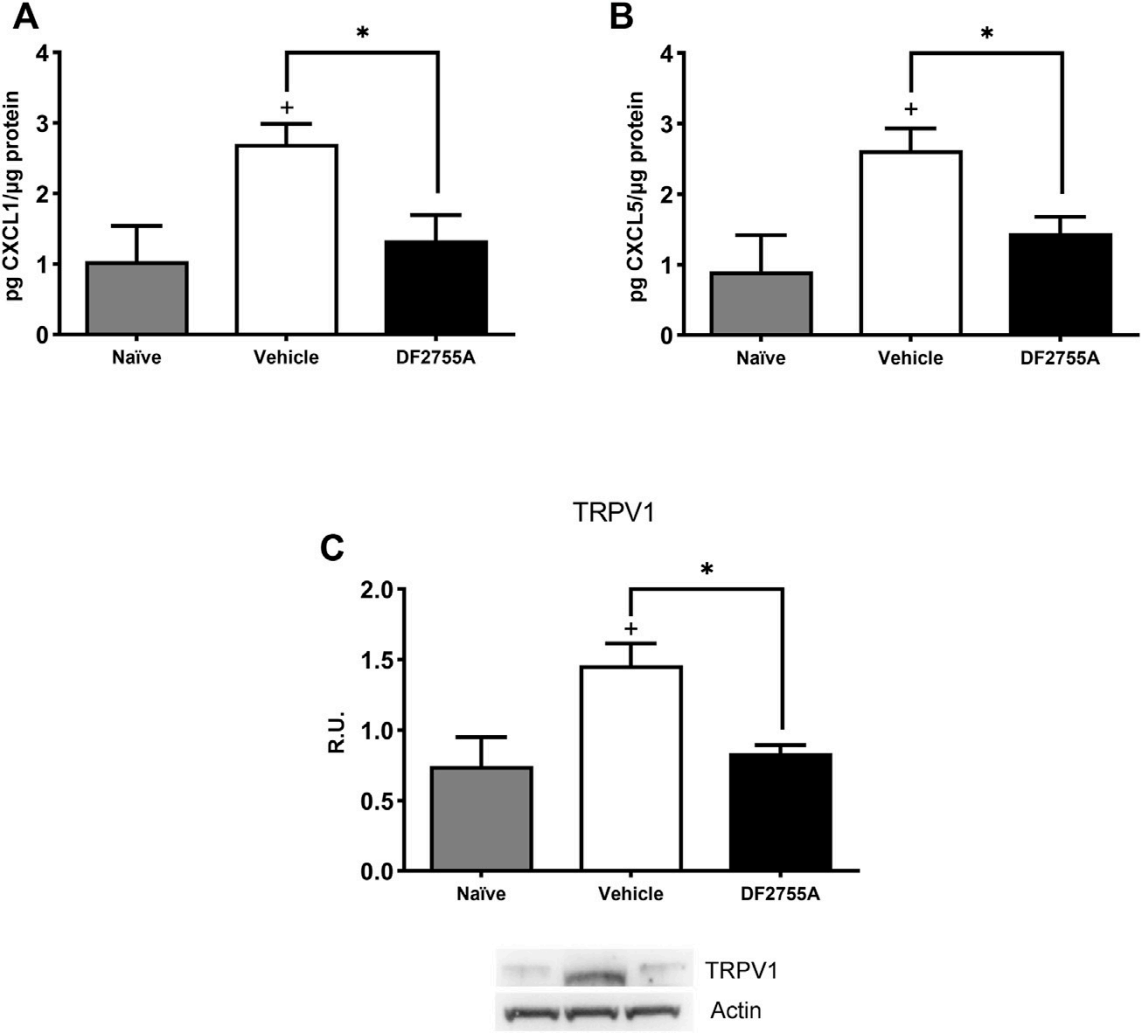
DF2755A preventive treatment inhibited CXCL1/CXCL5 production and TRPV1 receptor expression in IC bladder tissue. Rats were orally pretreated with repeated vehicle or DF2755A (20 mg kg^−1^ 12 h before the first CYP injection +7 mg kg^−1^ twice daily for 7 days) administrations. After 24 h of the last CYP injection, animals were killed, and bladders were removed and processed as described in Methods. CXCL1 **(A)** and CXCL5 **(B)** protein expression in whole urinary bladder tissue was determined with ELISAs in naïve rats, CYP- plus vehicle-treated rats (vehicle), and CYP- plus DF2755A-treated rats (DF2755A). Data are expressed as pg µg^−1^ of protein (mean values ± SEM; *n* = 5 per group). TRPV1 receptor expression was evaluated in bladder homogenates by Western blotting analysis and relative densitometric analysis as described in Methods. A representative band is reported **(C)**. Data are expressed as relative units (R.U.; mean values ± SEM; *n* = 5 per group). *p < 0.05 versus Vehicle group, +*p*< 0.05 versus Naïve group (ANOVA one-way).

To investigate the underlying mechanisms involved in DF2755A antinociceptive effects in CYP-induced peripheral neuropathy, we analyzed bladder tissue expression of the chemokines CXCL1/KC and CXCL5, which are CXCR2 agonists, and of transient receptor potential vanilloid 1 (TRPV1), a factor known to be crucial for pain modulation in IC/BPS pathophysiology (Z.Y. Wang ZY. et al., 2008). ELISA analysis showed that DF2755A treatment strongly inhibited bladder tissue expression of CXCL1/KC and CXCL5 compared to that observed in vehicle-treated rats (Figures 5A,B), while Western blot analysis revealed that DF2755A treatment also reduced TRPV1 bladder expression compared to vehicle-treated animals (Figure 5C). These data suggest that the antinociceptive effects of DF2755A can be due to its inhibition of the CYP-induced upregulation of chemokine signaling passing through CXCL1/CXCR1-2 axis and TRPV1.

**FIGURE 6:**
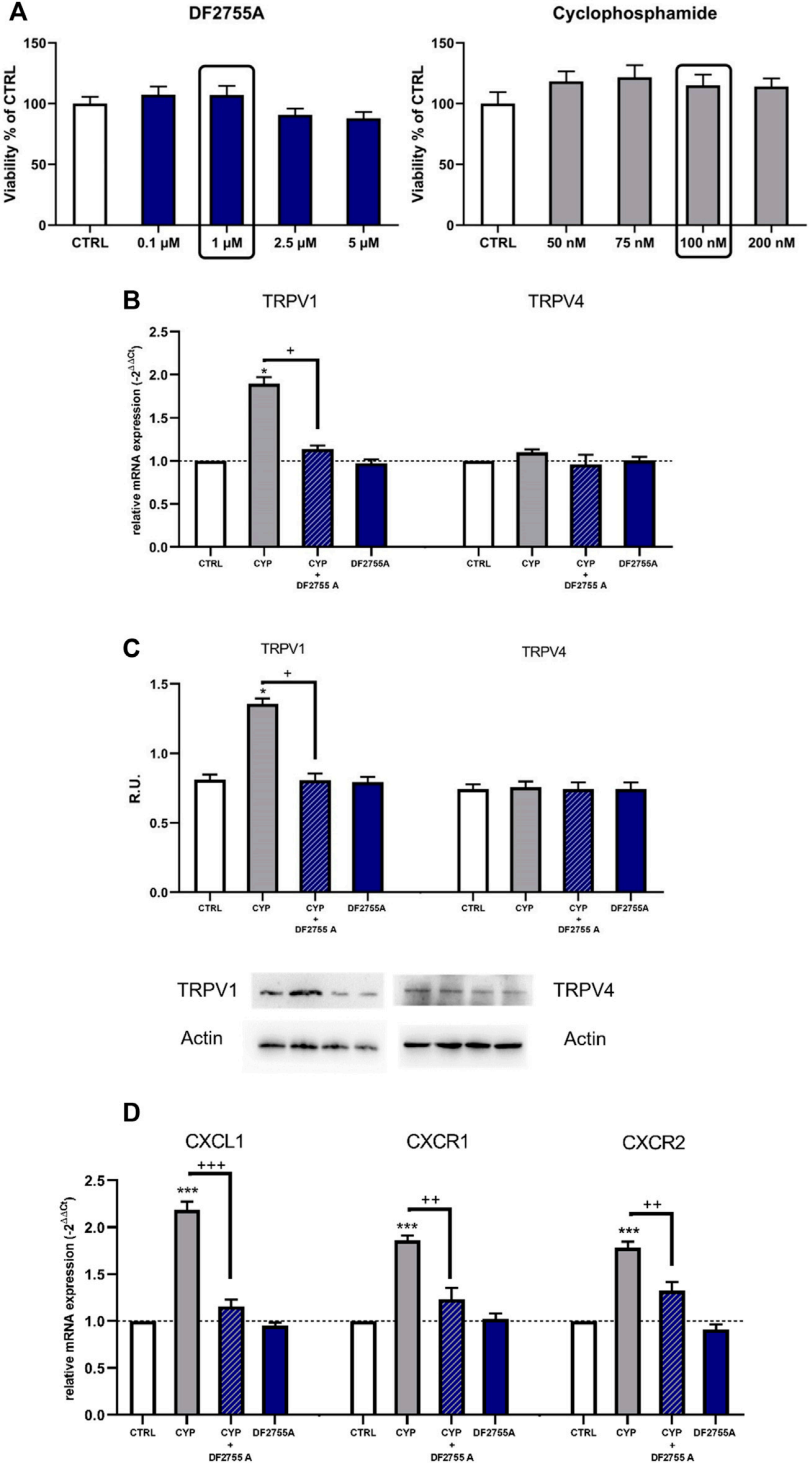
Expression of TRPV1, TRPV4, CXCL1, and its receptors CXCR1/2 upon treatment with CYP, DF2755A, and their combination was evaluated in the F11 cell line. F11 cells were exposed to different concentrations of DF2755A (0.1–5 µM) and CYP (50–200 nM) **(A)**; TRPV1 and TRPV4 expression in F11 cells upon treatment with CYP, DF2755A, and their combination was evaluated by RT PCR and Western blotting analysis and relative densitometric analysis **(B,C)**. A representative band is reported **(C)**. Data are expressed as relative units (R.U.; mean values ± SEM; *n* = 5 per group). Expression of CXCL1 and its receptors CXCR1/2 was evaluated by RT PCR in F11 cells upon treatment with CYP, DF2755A, and their combination **(D)**. *p < 0.05 and ***p < 0.0001 versus CTRL group; +*p*< 0.05 and +++*p*< 0.0001 versus CYP group (ANOVA one-way).

To confirm the direct modulation of TRPV1 expression by CYP and DF2755A, we investigated TRPV1 expression also *in vitro* in F11 cells, a validated model of dorsal root ganglion (DRG) sensory neurons (Haberberger et al., 2020), which are known target of chemokine-induced excitation mediated by TRPV1 (White et al., 2007). The cell viability assay showed that CYP at concentrations between 5 and 200 nM and DF2755A at concentrations between 0.1 and 5 µM did not modulate neuronal cell viability (Figure 6A); thus, we used DF2755A at a final concentration of 1 µM and CYP at a final concentration of 100 nM for the subsequent experiments. F11 cells exposed to CYP showed a significantly increased TRPV1 mRNA expression, and this effect was counteracted by treatment with DF2755A, which restored TRPV1 mRNA expression to control values (Figure 6B). On the other hand, TRPV4 mRNA expression did not change upon CYP stimulation. Western blotting analysis for TRPV4 and TRPV1 expression confirmed mRNA data showing an increased TRPV1 protein level upon CYP stimulation that was restored to control expression by the treatment with DF2755A (Figure 6C). Finally, to further investigate and validate the *in vivo* data, we analyzed also the expression of CXCL1 and of its receptors CXCR1 and CXCR2 in the F11 cell line. Results of these experiments showed that F11 cells exposed to CYP had a significantly increased mRNA expression of CXCL1, as well as of both its receptors CXCR1 and CXCR2 (Figure 6D). The upregulation of CXCL1-CXCR1/2 axis induced by CYP was effectively counteracted by the treatment with DF2755A, which restored the mRNA expression of all the targets to control values (Figure 6D).

Altogether, these results indicate that DF2755A treatment can directly modulate the increased expression of CXCL1-CXCR1/CXCR2 axis and TRPV1, and this modulation can be involved in its antinociceptive effect in this model of CYP-induced non-ulcerative IC.

## 4 Discussion

IC/BPS is a chronic disorder that severely impairs the quality of life of patients (Vasudevan and Moldwin 2017). While treatments for ulcerative (Hunner type) IC are available and provide good to excellent clinical results (Whitmore et al., 2019), limited response to pharmacological treatments has been observed in patients with non-ulcerative (non-Hunner type) IC with poor overall outcomes (Gupta et al., 2015; Whitmore et al., 2019). The present study demonstrates that oral treatment with DF2755A, a potent and selective inhibitor of chemokine receptors CXCR1/2, can prevent and reverse the peripheral neuropathy associated to CYP-induced non-ulcerative IC/BPS, exerting its antinociceptive effects by inhibiting chemokine-induced excitation of sensory neurons.

The role of chemokines in neuropathic pain has been associated mainly with their activities in the context of immune activation (Brandolini et al., 2017) and, in particular, with their functions in the recruitment and activation of leukocytes (White et al., 2007). However, increasing evidences are now extending the role of chemokines in chronic pain beyond their involvement in leukocyte recruitment and activation, suggesting that these molecules can also act directly on their receptors expressed by neurons, inducing chronic hyperexcitability and alterations in gene expression, which ultimately result in an abnormal and enhanced transmission of pain signals (White et al., 2007). Starting from these concepts, we investigated whether the inhibition of chemokine receptors CXCR1/2 by our compound DF2755A (Lopes et al., 2016) could reduce peripheral neuropathy in a rat model of CYP-induced non-Hunner IC/BPS, which is commonly characterized by the absence of inflammatory infiltrate (Augé et al., 2020).

DF2755A has an optimized pharmacokinetic profile and an extreme selectivity of action against a series of adrenergic, histaminic, muscarinic, neurokinin, opioid, serotoninergic, cannabinoid, and dopaminergic receptors as well as ion channels. These features, in association with its optimized oral bioavailability (F% 86.2), a half-life of 6.8 h, and distribution volume of 0.35 L/kg, make DF2755A an appropriate candidate for the treatment of painful conditions. In previous studies, we tested the effects of DF2755A in the treatment of inflammatory pain in mice (Lopes et al., 2016) and demonstrated that its analgesic effect in this condition was associated with the inhibition of CXCLs/CXCR1/2-mediated neutrophils recruitment and activation, a recognized pathway participating in inflammatory pain development (Cunha et al., 2008; Carreira et al., 2013). In the present study, we used a model of neuropathic pain and showed that DF2755A exerted a strong antinociceptive effect against peripheral neuropathy induced by chronic CYP administration in rats also in the absence of inflammatory infiltrate and that its anti-hyperalgesic effect lasted up to 7 days after treatment discontinuation.

The role of CXCR2 in the bladder function and visceral hypersensitivity in CYP-induced cystitis and the antinociceptive effect of a selective antagonist of CXCR2, SB225002, have been recently reported in another CYP-induced IC rat model (Dornelles et al., 2014). However, in that study the efficacy of CXCR2 inhibition was observed in an acute CYP-induced IC model, which was characterized by inflammatory bladder infiltrate and histopathologic alterations that are typical of ulcerative (Hunner type) IC. As a result, the antinociceptive effect of SB225002 was associated with the inhibition of bladder neutrophils infiltration, and the co-administration of a TRPV1 channel antagonist was necessary to obtain a complete counteraction of bladder inflammation and overactivity and of the nociceptive behavior (Dornelles et al., 2014). On the other hand, in our model of non-Hunner IC/BPS, DF2755A alone as preventive or therapeutic treatment was strikingly protective against CYP-induced peripheral neuropathy, thus demonstrating that CXCR2 plays also a crucial role in the pathophysiology of IC-related neuropathic pain that is independent from tissue inflammatory infiltrate.

Analyzing the mechanisms underlying DF2755A antinociceptive action, we found that its therapeutic effects were associated with a strong reduction of chemokines CXCL1/KC and CXCL5 and TRPV1 expression *in vivo* and in a model of sensory neurons *in vitro*. These results are in line with the idea that CXCLs chemokines and their CXCR1/R2 receptors are directly involved in the sensitization of primary nociceptive neurons, and that this is independent from their roles in inflammation. In fact, numerous studies have suggested a direct pro-nociceptive role for CXCL1 in the peripheral nervous system and demonstrated that CXCL1 is crucially involved in the modulation of pain hypersensitivity (Qin et al., 2005; Zhang et al., 2013). Intra-plantar or intra-articular injection of CXCL1 produced mechanical hyperalgesia in rodents (Qin et al., 2005; Guerrero et al., 2012), while the spinal injection of exogenous CXCL1 induced heat hyperalgesia and activation of spinal cord neurons through CXCR2 (Zhang et al., 2013). Interestingly, behavioral tests showed that the intrathecal administration of CXCL1 neutralizing antibody induced a dose-dependent inhibition of spinal nerve ligation-induced pain hypersensitivity, suggesting a key role of the CXCL1/CXCR2 axis in neuropathic pain sensitization (Zhang et al., 2013).

One of the mechanisms through which CXCL1/CXCR1/2 axis activation could induce nociceptive neurons excitability is the activation of ion channels, such as TRPV1, which are known to crucially contribute to the development and maintenance of pain (Z.-Y. Wang ZY. et al., 2008). The TRPV1 channel is in fact expressed by not only afferent nerve fibers that innervate the bladder (Ost et al., 2002) but also DRG neurons, on which CXCL1 has been shown effective in directly modulating neuronal excitability by increasing sodium currents, potassium currents, and the function of TRPV1 channels (Zhang et al., 2013; Verri et al., 2006; J.-G. ; Wang JG. et al., 2008). In light of these studies and of our data, we can thus suggest that the antinociceptive effects that we observed after the treatment with DF2755A can be due to its inhibition of the CXCL1/CXCR1-2 axis, which results in turn in the inhibition of pain-related proteins as TRPV1. Notably, our results are even more significant since we used a model of IC induced by CYP, a chemotherapeutic agent that is known to induce peripheral neuropathy. Chemotherapy-induced peripheral neuropathy is a common and disabling side effect of many anti-cancer drugs (Cavaletti and Marmiroli 2010), often resulting in treatment discontinuation, which may ultimately affect overall survival (Markman 1996). Thus, in addition to its demonstrated effects in counteracting non-Hunner IC/BPS, we can hypothesize that DF2755A might also exert antinociceptive effects in the treatment of CYP-induced peripheral neuropathy. Further studies will be aimed at more deeply investigating also the molecular effects of the treatment with DF2755A on nociceptors in this model.

In conclusion, the data reported in the present article demonstrate that the inhibition of CXCR1/2 through DF2755A can be a potential therapeutic approach for the treatment of not only inflammatory painful conditions but also neuropathic ones and in particular peripheral neuropathy in non-Hunner IC/BPS.

## Data Availability

The original contributions presented in the study are included in the article/Supplementary Material, further inquiries can be directed to the corresponding author.
